# Therapeutic microRNAs: Mechanisms, Delivery, and Clinical Translation in Oncology

**DOI:** 10.3390/ijms27052162

**Published:** 2026-02-25

**Authors:** Humberto Vélez-Slimani, Luis A. Salazar

**Affiliations:** 1 Doctoral Program in Sciences Major in Applied Cellular and Molecular Biology, Universidad de La Frontera, Temuco 4811230, Chile; h.velez01@ufromail.cl; 2 Center for Molecular Biology and Pharmacogenetics, Department of Basic Sciences, Universidad de La Frontera, Temuco 4811230, Chile

**Keywords:** microRNA therapeutics, miRNA delivery, oligonucleotide chemistry, extracellular vesicles, clinical translation, target engagement

## Abstract

MicroRNAs (miRNAs) are ~19–25-nt post-transcriptional regulators whose dysregulation promotes hallmark cancer traits and therapy resistance. This review synthesizes translational principles for developing miRNA therapeutics in oncology, integrating miRNA biology and target engagement with delivery design and clinical experience. We summarize key determinants that shape efficacy and safety, including sequence and chemistry choices, biodistribution and intracellular delivery, dosing strategy, and biomarker-informed patient selection. We compare the main therapeutic modalities, miRNA mimics and inhibitors, and evaluate leading delivery approaches relevant to cancer, including lipid-based systems, polymer-based carriers and conjugates, and extracellular vesicle-inspired platforms, highlighting trade-offs in stability, specificity, immune activation, and tumor exposure. Early clinical programs such as MRX34, TargomiR/MesomiR-1, and cobomarsen, together with experience from non-oncology indications, illustrate both opportunities and practical constraints on tolerability and regimen optimization. We conclude with pragmatic priorities for the field, including standardized analytics for isoforms and target engagement, PK/PD- and biomarker-guided dose selection, and rational combination strategies to safely integrate miRNA-based interventions into precision oncology.

## 1. Introduction

Widespread transcription and rich catalogs of candidate regulatory elements from the ENCODE project have highlighted the central roles of non-coding RNAs (ncRNAs) in gene regulation across cell types and states [1,2]. Phase III of ENCODE expanded multi-omic maps of transcription, chromatin, and RBP (RNA-binding proteins) occupancy across human and mouse tissues [2]. MiRNAs are small ncRNA molecules that play a critical role in gene expression regulation by binding to target mRNAs to inhibit protein translation or initiate mRNA degradation. Functionally, they typically guide Argonaute to partially complementary sites, most often within 3′-UTRs, enforcing translational repression and accelerating mRNA decay [3]. The most effective canonical site types, 8mer, 7mer-m8, and 7mer-A1, typically reside in 3′-UTRs, although CDS (coding sequence) and even 5′-UTR sites can contribute in specific contexts [4,5].

**Figure 1:**
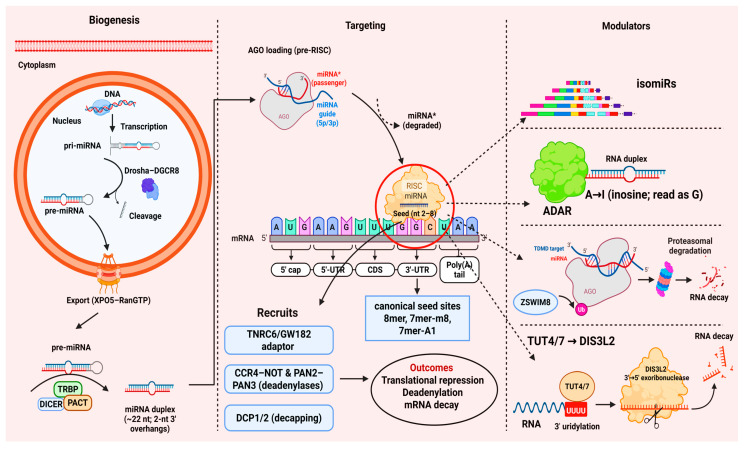
The microRNA lifecycle. Biogenesis: In the nucleus, RNA Pol II transcribes pri-miRNAs, which are cropped by the Drosha–DGCR8 microprocessor to pre-miRNAs and exported to the cytoplasm by Exportin-5/RanGTP. Dicer, with TRBP/PACT, cleaves pre-miRNAs into approximately 22-nt duplexes with 2-nt 3′ overhangs. Targeting: Argonaute loading (pre-RISC) selects the guide strand and discards the passenger (miRNA*). The AGO/miRISC then binds complementary sites primarily in 3′-UTRs, especially canonical seed sites (miRNA nt 2–8): 8mer, 7mer-m8, and 7mer-A1 (sites in CDS/5′-UTR can contribute context-dependently). Repression & Decay: Target binding recruits TNRC6/GW182, which engages the CCR4–NOT and PAN2–PAN3 deadenylases and the DCP1/2 decapping complex, coupling translational repression to deadenylation and mRNA decay. Modulators: isomiRs (processing variants), ADAR A → I editing, TDMD (target-directed miRNA degradation) via the ZSWIM8 E3 ligase (AGO ubiquitination → proteasome), and terminal uridylation by TUT4/7 that promotes DIS3L2-mediated 3′ → 5′ degradation refine targeting efficacy and miRNA stability. Solid arrows indicate the main pathway; dashed arrows indicate modulatory influences on targeting. AGO—Argonaute; miRISC—miRNA-induced silencing complex; UTR—untranslated region; CDS—coding sequence; TDMD—target-directed miRNA degradation; XPO5—Exportin-5. Created in BioRender. Vélez Slimani, H. (2025) https://BioRender.com/j7azj0w (accessed on 31 October 2025).

The biogenesis of miRNAs involves several steps. It begins with transcription, followed by nuclear processing by the Drosha-DGCR8 complex, cytoplasmic cleavage by Dicer, and the incorporation of mature miRNA duplexes into RNA-induced silencing complexes (RISCs), which mediate gene silencing through interactions with target mRNAs [6]. In metazoans, Argonaute-loaded miRNAs recruit GW182/TNRC6, which engages the CCR4–NOT and PAN2–PAN3 deadenylases and decapping factors, coupling translational repression to mRNA decay [7,8]. Processing heterogeneity generates isomiRs that can shift seed usage; A-to-I editing by ADARs can retarget mature species; and highly complementary sites can trigger target-directed miRNA degradation (TDMD) via the ZSWIM8 ubiquitin ligase, mechanisms that shape targeting and steady-state abundance [9,10,11,12]. The selective loading of miRNAs into extracellular vesicles supports intercellular communication, but rigorous interpretation under MISEV2023 guidelines is essential [13] (Figure 1).

Some miRNAs are overexpressed, promoting oncogenic processes, while others are downregulated, acting as tumor suppressors [14]. In recent years, miRNAs have emerged as promising biomarkers for cancer diagnosis and therapeutic targets, enabling personalized medicine by tailoring treatments to individual genetic profiles [15]. This duality of tumor-suppressive and oncogenic miRNAs is well established in cancer biology [14].

RNA-based therapies, particularly those using miRNAs, emerge as a promising alternative to address challenges in oncology [16]. These therapies leverage the dual role of miRNAs in cancer by targeting oncogenic miRNAs or tumor suppressors to restore balance. Overexpressed miRNAs can promote tumor progression, while downregulated miRNAs fail to inhibit malignant growth. Strategies include miRNA mimics to restore suppressor functions or inhibitors (antimiRs) to neutralize overexpressed oncogenic miRNAs. Due to their small size and chemical properties, miRNAs can be easily synthesized and packaged into delivery systems targeting tumor cells, potentially reducing the side effects of conventional treatments. These therapies offer the potential to personalize treatment based on the miRNA profiles of tumors, enhancing therapeutic efficacy and reducing adverse effects [15]. Recent overviews highlight key clinical programs (e.g., MRX34, anti-miR-122, anti-miR-155) and emphasize delivery and innate-immune activation as central translational hurdles [17].

Here, we provide a critical and clinically oriented synthesis of therapeutic microRNAs in oncology. We summarize miRNA biogenesis and target recognition, highlight context-dependent roles across tumor and immune compartments, review delivery strategies with an emphasis on nanotechnology, and discuss lessons from early clinical programs and the main translational challenges.

## 2. Biogenesis and Mechanism of Action of microRNAs

MicroRNAs originate from long primary transcripts (pri-miRNAs) that, within the nucleus, are processed by the Microprocessor complex, comprising the RNase III enzyme DROSHA and its cofactor DGCR8, into precursor molecules (pre-miRNAs) of approximately 60–80 nucleotides. These pre-miRNAs are subsequently transported to the cytoplasm by Exportin 5, where the RNase III enzyme Dicer further cleaves them to produce mature microRNAs [18]. The Microprocessor exhibits structural flexibility, accommodating diverse pri-miRNA architectures while preserving cleavage fidelity [19]. High-throughput and structural studies indicate that short sequence and local-structure cues in pri-miRNAs help position Drosha and improve cleavage precision and productive processing (e.g., basal and downstream elements and lower-stem features described for subsets of substrates), and these concepts are summarized for quick reference in Table 1 [20,21,22,23]. Key terms and selected non-canonical routes mentioned in this section are summarized in Table 1 to improve accessibility for non-specialist readers.

Position-specific defects in the lower stem, including internal loops and asymmetric bulges, can inhibit productive Microprocessor cleavage and increase alternative or unproductive cuts, thereby lowering mature miRNA yield in a context-dependent manner [24,25]. Beyond hairpin-intrinsic cues, RNA-binding proteins add cell-type-specific control. In high-throughput pri-miRNA cleavage assays, SRSF7/SRSF3 recognize sequence/structure motifs (e.g., CNNC) to enhance or redirect Drosha cleavage at selected substrates, refining Microprocessor selectivity [26].

DGCR8 not only acts as a cofactor of Drosha but can also recognize specific RNA structural elements in certain pri-miRNAs, such as pri-miR-9-2. This recognition involves a conserved DGCR8-responsive RNA element (DRE) that enhances the efficiency of pri-miRNA processing. Such sequence- and structure-dependent selectivity highlights that not all pri-miRNAs are processed with the same efficiency, adding an additional regulatory layer to microRNA biogenesis [27]. In pri-miR-9-2, a conserved DGCR8-responsive RNA element (DRE) confers heightened DGCR8 sensitivity and may be tuned by epitranscriptomic modifications, providing a mechanistic basis for selective cofactor engagement [27]. Processing heterogeneity generates isomiRs via alternative Drosha/Dicer cleavage and 3′ non-templated additions, which can shift seed usage and alter target selection and stability. In parallel, A-to-I RNA editing by ADAR1/ADAR2 in pri/pre-miRNAs can redirect processing and retarget mature miRNAs, with disease-relevant consequences documented in human tissues and cancer. Together, isomiR diversity and editing add a regulatory layer to miRNA biogenesis and targeting that should be considered when interpreting expression and function [28,29,30].

### 2.1. Non-Canonical Pathways

Beyond the canonical Drosha–DGCR8/Dicer route, several non-canonical modes generate functional miRNAs. Mirtrons arise from short introns that, after splicing, form a lariat and are then debranched by DBR1 into pre-miRNA-like hairpins that enter Exportin-5/Dicer; in vertebrates, RNA G-quadruplexes (rG4s) positioned in the 5′ arm of mirtrons promote splicing and maturation, adding a structural checkpoint to their biogenesis. miR-451 is the prototypical Dicer-independent miRNA: its pre-hairpin is sliced by AGO2 and subsequently 3′ trimmed by PARN; recent work shows CSDE1 binds pre-miR-451 and facilitates AGO2 processing and PARN trimming during erythropoiesis. In addition, tRNA-derived small RNAs (tsRNAs/tRFs) and snoRNA-derived fragments (sdRNAs) can, in specific contexts, load into Argonaute and exert miRNA-like regulation, expanding the small-RNA regulatory landscape beyond canonical precursors [31,32,33,34].

### 2.2. RISC Loading and Gene Silencing

After Dicer cleavage, the miRNA duplex is loaded onto AGO with assistance from TRBP/PACT and the Hsc70/Hsp90 chaperone machinery; duplex asymmetry and 5′-end thermodynamic stability bias guide-strand selection. Activated AGO engages TNRC6/GW182, which recruits the CCR4–NOT and PAN2–PAN3 deadenylases together with decapping factors to drive mRNA decay alongside translational repression [3,35,36,37,38].

Beyond repression and decay of target mRNAs, miRNA abundance itself is actively sculpted by target-directed miRNA degradation (TDMD). Highly complementary binding sites trigger a conformational change in AGO that recruits the ZSWIM8 E3 ubiquitin ligase, leading to AGO ubiquitination and rapid miRNA decay, largely independent of 3′ tailing/trimming. TDMD operates on cellular and viral transcripts and is prominent in neurons and immune cells; accounting for TDMD kinetics is therefore crucial when interpreting miRNA steady-state levels and when designing mimics or antimiRs for therapy [9,10]. Complementing TDMD, a broader uridylation-coupled decay program sculpts miRNA abundance: TUT4/TUT7 add 3′-uridylation to select pre-/mature miRNAs, targeting them for DIS3L2-mediated degradation; converging structural and genomic data support uridylation as the main 3′ mark impacting global steady-state levels [39,40].

In mammalian cells, miRNA-mediated repression frequently initiates on rough-ER-bound polysomes, and while multiple AGO paralogs participate, AGO2 is the catalytically robust “slicer” (with AGO1/3/4 primarily mediating non-endonucleolytic repression under physiological conditions) [41].

### 2.3. Seed Sequence and Targeting Rules

Additionally, target recognition by miRNAs is primarily mediated through a “seed sequence” comprising nucleotides 2–8 from the 5′ end of the miRNA, which pairs with complementary sites in the 3′ untranslated region (3′-UTR) of target mRNAs [42]. Many miRNA loci map within genomic regions prone to structural variation (e.g., fragile sites, recurrent copy-number changes or LOH in cancer), supporting a link between locus instability and disease susceptibility, although prevalence varies by dataset and tumor type [43]. Functionally validated site types follow an affinity hierarchy (8mer > 7mer-m8 ≈ 7mer-A1 > 6mer), and extensive 3′-supplementary/compensatory pairing can rescue weak seeds; high-throughput binding and reporter assays quantitatively capture these rules. Moreover, while most effective sites reside in 3′-UTRs, CDS and 5′-UTR interactions are increasingly documented by AGO-CLIP/eCLIP-chimera studies and can contribute to repression in a context-dependent manner [44,45].

### 2.4. Exosomal miRNA Communication

Beyond these intracellular pathways, microRNAs can be selectively packaged into exosomes, small extracellular vesicles ranging from 30 to 150 nm in diameter, and secreted into the extracellular environment. These exosomes can be taken up by neighboring or distant cells, where the transferred microRNAs modulate gene expression and influence processes such as proliferation, angiogenesis, invasion, metastasis, and drug resistance. This intercellular transfer mechanism extends the functional reach of microRNAs beyond the cells in which they are originally produced [46]. Selective loading of miRNAs into small extracellular vesicles involves RNA-binding proteins such as YBX1 and hnRNPA2B1, which bind sequence/structure motifs and can phase-separate into P-body-like condensates that interface with vesicle biogenesis. The field-wide MISEV2023 consensus updates isolation/characterization criteria and cautions against over-interpreting low-copy miRNA cargo without rigorous controls [13,47,48]. To better separate high-confidence EV-miRNA studies from less rigorous reports, it is important to align interpretation with MISEV2023. In most settings, “small extracellular vesicles (sEVs)” is the more accurate term unless endosomal origin is directly demonstrated. High-confidence evidence generally includes an isolation workflow that minimizes co-isolated non-vesicular carriers (for example, size-exclusion chromatography and/or density-based separation rather than precipitation alone), careful EV characterization using recommended positive and negative markers together with particle sizing/counting, and RNase-based controls performed with and without detergent to support true encapsulation. These practices should be complemented by transparent quantitative reporting and normalization (including particle numbers and, where feasible, estimates of miRNA copies per vesicle), as well as functional transfer assays with appropriate EV-depleted and dose–response controls. Studies relying mainly on precipitation kits, limited marker panels, or unclear normalization should be interpreted cautiously, especially given evidence that many extracellular miRNAs are abundant in non-vesicular Argonaute complexes and that average miRNA copy numbers per exosome can be low.

## 3. Role of miRNAs in Cancer Pathogenesis

This section outlines how miRNAs contribute to cancer hallmarks across malignant, immune, and stromal compartments, and prepares the ground for therapeutic strategies discussed in Section 4.

### 3.1. General Mechanisms of Dysregulation in Cancer

Aberrant expression of certain non-coding RNAs, including miRNAs, contributes to the development and progression of multiple cancers, acting as potential biomarkers and therapeutic targets. Over the past decade, around 20 clinical trials have been initiated to evaluate miRNA-based therapies, with some reaching phase II for advanced cancers, such as TargomiR (miR-16), Cobomarsen (anti-miR-155), and Miravirsen (anti-miR-122) [49]. Among the most extensively studied microRNAs, miR-34a-5p has attracted considerable attention as a tumor-suppressive RNA that regulates multiple oncogenic pathways. However, its clinical translation has been hindered by challenges related to stability, delivery, and toxicity, prompting the exploration of novel strategies to harness its therapeutic potential [50]. Dysregulation arises from altered biogenesis (DROSHA/DICER/XPO5), genomic/epigenetic changes (amplifications, deletions, promoter methylation), and post-processing modifiers (isomiRs, ADAR editing, TDMD, terminal uridylation).

### 3.2. OncomiRs and Tumor-Suppressor miRNAs

A large proportion of human genes are regulated by miRNAs, leading to miRNA-dependent modulation of several essential cellular processes, including differentiation, survival, proliferation, and cell death [51]. Thousands of human miRNA loci give rise to several thousand mature miRNAs, collectively regulating a substantial fraction of the transcriptome. While many have well-defined functions, numerous miRNAs remain incompletely characterized. In the context of cancer, miRNAs may function as either oncogenes or tumor suppressors, and their dysregulation has been associated with key tumorigenic processes, including proliferative signaling, cellular invasion, angiogenesis, and metastasis [52].

In cancer, miRNAs function as oncogenic (oncomiRs) or tumor-suppressive regulators, modulating key pathways (PI3K/AKT, EMT, angiogenesis, immune evasion) by silencing multiple targets. Among oncomiRs, miR-21 is prototypical, downregulating PTEN and PDCD4 to promote proliferation, invasion, and survival [53]. miR-155 drives progression by suppressing SOCS1/SHIP1/TP53INP1 and reshaping the tumor microenvironment [54]. miR-10b facilitates metastasis through HOXD10 inhibition and consequent RhoC de-repression [55]. miR-221/222 reduce p27Kip1, PTEN, and TIMP3, sustaining proliferation and invasion [56]. On the suppressive side, miR-34a-5p constrains oncogenic networks (p53/MET/CDK6), though clinical translation has been limited by stability, delivery, and toxicity issues (e.g., MRX34) [57]. The let-7 family represses RAS (Rat sarcoma proto-oncogene, small GTPase signaling) and HMGA2 (High Mobility Group AT-hook 2), restricting proliferation (cell growth/division) and EMT (epithelial–mesenchymal transition) [58]. The miR-15a/16-1 cluster antagonizes BCL2 and promotes apoptosis, with foundational relevance in CLL (chronic lymphocytic leukemia) [59]. miR-143/145 are frequently downregulated in colorectal and other tumors; restoration curbs invasion and pro-tumor stroma [60]. Finally, the miR-200 family suppresses EMT by inhibiting ZEB1/ZEB2, maintaining epithelial identity and limiting metastasis (the miR-200/ZEB feedback loop) [61]. (see Table 2).

The role of miRNAs in cancer is complex due to their ability to target multiple genes within different pathways. Beyond tumor-intrinsic effects, miRNAs also rewire immune and stromal interactions within the tumor microenvironment.

### 3.3. Immuno-Oncology and the Tumor Microenvironment

miRNAs orchestrate the tumor microenvironment by reprogramming immune and stromal cues. Pro-inflammatory miR-155 and immunoregulatory miR-21/miR-146a modulate macrophage polarization (M1/M2), thereby shaping antitumor versus protumor programs [62]. Tumor-derived exosomal miRNAs refine immune evasion and angiogenesis; for example, exosomal miR-1246 stabilizes PD-L1 and suppresses CD8^+^ T-cell activity, while other exosomal miRNAs interface with VEGF pathways and EMT to promote metastatic dissemination [63]. Macrophage–tumor cross-talk mediated by exosomal miRNAs (e.g., miR-21-5p from M2 macrophages) has been linked to enhanced invasion and therapy resistance, underscoring the translational need for stabilized chemistries and targeted delivery [64] (see Table 3).

### 3.4. Immune Regulation and Delivery Constraints

In recent years, multiple studies have demonstrated the involvement of microRNAs (miRNAs) in regulating macrophage polarization, positioning them as emerging therapeutic targets. However, the rapid degradation of miRNAs and their synthetic analogs, mimics (agomirs) and inhibitors (antagomirs), in biological fluids, together with their ability to modulate multiple transcripts and signaling pathways, limits their clinical applicability. These challenges, combined with the need to improve stability, specificity, and delivery systems, have slowed their translation into clinical practice [65,66].

### 3.5. Clinical Translation and Early Trials

Most first-in-human microRNA therapeutic programs follow a conventional early-phase framework in heavily pretreated populations. Primary objectives focus on safety and tolerability, including dose-limiting toxicities, maximum tolerated dose, and the recommended phase 2 dose. Secondary objectives include pharmacokinetics and pharmacodynamics, evidence of target engagement, and preliminary clinical activity such as response or disease control. Across programs, discontinuation has reflected diverse drivers, including immune-mediated toxicities, strategic or business considerations, and insufficient efficacy at tolerable exposure. Table 4 summarizes the clinical-stage microRNA therapeutics discussed in this review, reporting trial phase/status, indication, delivery platform/route, key chemistry/modifications, and outcomes/limitations.

Beyond listing clinical programs, the clinical performance of miRNA therapeutics is shaped by multiple variables that extend beyond mechanistic rationale. Sequence and oligonucleotide design can influence potency while constraining seed-driven off-targeting, and chemical modification patterns can shift the balance between stability, exposure, and innate immune activation. Delivery platform selection strongly affects biodistribution, tumor penetration, and endosomal escape, which in turn can determine whether intracellular activity is achieved at tolerable systemic exposure. Clinical outcomes are also conditioned by dosing strategy, including dose intensity, infusion schedule, and prophylactic measures that can mitigate inflammatory reactions. Finally, patient-related factors such as tumor target expression, baseline immune status, and prior lines of therapy can modulate exposure–response relationships and the likelihood of adverse events. This framework helps interpret why early programs could show pharmacodynamic signals yet still be limited by tolerability and supports more rational trial design.

Despite these constraints, several candidates entered early testing. MRX34 (miR-34a mimic) was evaluated in advanced tumors (NCT02862145, NCT01829971) but was discontinued due to immune-related toxicities. This experience suggested that tolerability can be dominated by formulation- and regimen-dependent innate immune activation, particularly in heavily pretreated populations. It also highlighted the need to align sequence/chemistry choices with immunomonitoring and to optimize dosing and premedication to reduce inflammatory peaks while preserving target engagement. Subsequent preclinical work has revisited miR-34a using next-generation designs that emphasize extensive chemical stabilization and active targeting. In particular, a fully chemically modified miR-34a showed markedly improved nuclease stability while preserving activity, and ligand-directed delivery strategies using small-molecule targeting moieties have enhanced tumor selectivity and intracellular activity. Together, these studies provide a direct translational bridge between the MRX34 experience and current advances in miR-34a chemistry and targeted delivery. MesomiR-1 (miR-16 mimic) demonstrated tolerability in a phase I trial for malignant pleural mesothelioma and non-small-cell lung cancer. This case illustrates how carrier selection and targeting can improve feasibility, while regimen variables and baseline patient inflammation may still constrain dose intensity and exposure. Among antagomirs, SPC3649/Miravirsen (anti-miR-122) completed several phase II trials in hepatitis C, while RG-012/Lademirsen (anti-miR-21) and MRG-106/Cobomarsen (anti-miR-155) have been evaluated in renal and hematologic disorders, respectively. Other examples include MRG-110 (anti-miR-92a), RG-125/AZD4076 (anti-miR-103/107), RG-101 (anti-miR-122), and AMT-130 (miHTT), all still in early-phase development [73]. Prospectively, biomarker-guided patient selection and integrated PK/PD and immunomonitoring should inform dosing windows for miRNA mimics and antimiRs, explicitly linking target engagement and pharmacodynamic effects to clinical response and tolerability.

### 3.6. miRNAs in Cell Death and Resistance

MiRNAs regulate key processes in programmed cell death (PCD) in stem cells, such as differentiation and proliferation. miRNAs influence apoptosis, autophagy, and ferroptosis by interacting with signaling pathways like PI3K/Akt and TGF-β. Additionally, miRNAs have the potential to be biomarkers and therapeutic targets in stem cell therapy, although their clinical application faces challenges like precision in delivery and treatment heterogeneity [74]. Recent studies have shown that miRNAs play a crucial role in the development of drug resistance in various types of cancer [75]. Illustratively, miR-21 promotes cisplatin resistance via the PTEN/PI3K-AKT axis [76,77]; exosomal miR-221/222 has been linked to tamoxifen resistance in ER-positive breast cancer [78,79]; and restoring miR-200c can re-sensitize tumors to EGFR-TKIs by counteracting EMT-associated mechanisms [80,81].

### 3.7. microRNAs and Cancer Stem Cells (CSCs)

MiRNAs regulate CSC identity and plasticity by tuning Wnt/β-catenin, Hedgehog, Notch, JAK/STAT, TGF-β/SMAD, and PI3K/AKT/mTOR pathways, thereby sustaining self-renewal, therapy resistance, and immune evasion. The glycocalyx interface (e.g., CD44/hyaluronan) and biomarkers such as CD133, ALDH1, and EpCAM provide therapeutic entry points. Targeted inhibition or restoration of specific miRNAs, combined with CSC-directed approaches (e.g., CAR-T or lipid-metabolism blockade), aims to eliminate resistant clones. Single-cell omics, CRISPR screens, and patient-derived organoids enable mapping of miRNA–CSC networks and prioritization of translational targets [82]. Notably, miR-34a, let-7, and the miR-200 family intersect CSC programs and EMT, suggesting that restoring these axes, together with CSC-directed modalities (e.g., anti-CD44, CAR-T), may help deplete resistant clones [61,83,84,85]. Collectively, miRNAs coordinate cancer hallmarks across malignant, immune, and stromal niches, creating actionable liabilities, either by inhibiting oncomiRs or restoring tumor-suppressive miRNAs and setting the stage for delivery strategies detailed in Section 4.

## 4. Therapeutic Strategies and Delivery Platforms for miRNAs

**Figure 2:**
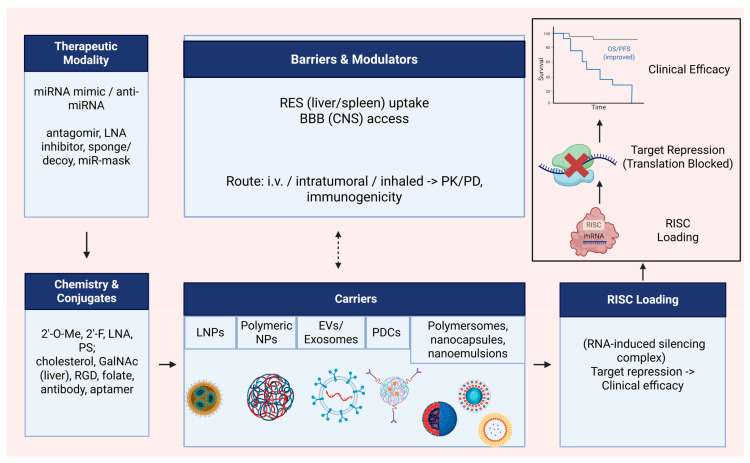
Determinants of successful miRNA delivery. Chemical stabilization (2′-O-Me, 2′-F, LNA, PS) and conjugates (cholesterol, GalNAc, RGD/folate/antibody/aptamer) enhance stability and targeting. Carriers: LNPs (ionizable + PEG/helper lipids), polymeric nanoparticles (PLGA/dendrimers/PEGylated), extracellular vesicles (EVs/exosomes), polymer–drug conjugates (PDCs), and other nanotechnologies (polymersomes/nanocapsules/nanoemulsions), govern biodistribution and endosomal escape, enabling RISC (RNA-induced silencing complex) loading. Barriers include RES (reticuloendothelial system) uptake and BBB (blood–brain barrier) for CNS delivery. PK/PD (pharmacokinetics/pharmacodynamics) and immunogenicity are shaped by route (i.v., intratumoral, inhaled) and composition. Grey line indicates baseline/no improvement, whereas the blue line indicates improved OS/PFS. Created in BioRender. Vélez Slimani, H. (2025) https://BioRender.com/c0kf54b (accessed on 31 October 2025).

Nucleic acid-based therapies have emerged as an innovative strategy to treat various conditions, using mRNA, siRNA, and miRNA to modulate specific genes and counteract severe diseases [86]. Nanotechnology-based delivery platforms offer significant potential to enhance the stability, bioavailability, and therapeutic efficacy of miRNA-based treatments. By enabling targeted delivery, protecting miRNAs from degradation, and improving cellular uptake, these systems, including lipid nanoparticles, polymeric carriers, exosomes, and conjugates, can optimize therapeutic outcomes while reducing off-target effects [87] (see Figure 2 for an overview of delivery determinants and barriers).

Therapeutic modalities include miRNA mimics (to restore tumor-suppressive miRNAs) and anti-miRNA agents (antagomirs, LNA inhibitors, sponges/decoys, miRNA masks) to inhibit oncomiRs; platform choice governs stability, biodistribution, endosomal escape, and on-/off-target effects [88,89].

### 4.1. Lipid Nanoparticles (LNPs)

Cross-cutting note: Chemical stabilization (2′-O-Me, 2′-F, LNA, phosphorothioate) and conjugates (e.g., cholesterol, GalNAc for liver, RGD/folate/antibody/aptamer ligands) enhance serum stability, cell uptake, and tissue targeting while reducing dose requirements [90,91]. Modern ionizable lipid nanoparticles (LNPs) and fusogenic/pH-responsive lipids improve endosomal escape, a key barrier for cytosolic RISC loading; helper lipids and PEG-lipids tune PK/PD and immunogenicity [92,93,94]. Administration route (intravenous, intratumoral, inhaled) critically shapes PK/PD, organ exposure (liver/spleen), and immunogenicity profiles [95,96].

### 4.2. Solid Lipid Nanoparticles (SLNs) and Nanostructured Lipid Carriers (NLCs)

Among them, solid lipid nanoparticles (SLNs) and nanostructured lipid carriers (NLCs) stand out for their ability to improve oligonucleotide stability, protect against enzymatic degradation, and enable controlled release, making them promising candidates for miRNA therapies. However, endosomal escape is typically less efficient than in LNPs and may require complementary strategies (e.g., fusogenic or pH-responsive lipids) [97].

### 4.3. Polymeric Carriers (PLGA, Dendrimers, PEGylated)

Polymeric nanocarriers, such as PEGylated nanoparticles, dendrimers, and biodegradable polymers (e.g., PLGA), offer controlled release, protection against nuclease degradation, and tunable surface modification for targeted delivery of miRNA therapeutics. Innovations include stimuli-responsive systems triggered by pH, enzymatic activity, or redox conditions within the tumor microenvironment, allowing precise spatiotemporal release. Functionalization strategies, such as PEGylation or ligand conjugation, can further extend circulation time, lower immunogenicity, and improve tumor accumulation [98]. Polymeric systems enable co-delivery (miRNA + chemo/kinase inhibitor) and TME-responsive release (pH, ROS, MMPs), improving tumor penetration and synergy with standard-of-care [99,100].

### 4.4. Extracellular Vesicles (EVs) and Exosomes

Tissue-derived extracellular vesicles, particularly exosomes, offer advantages such as biocompatibility, low immunogenicity, and inherent targeting capabilities, making them promising candidates for therapeutic RNA delivery [101]. When derived from stem cells, exosomes also exhibit regenerative and immunomodulatory properties. Their membranes, enriched with adhesion and signaling proteins, facilitate uptake by target cells, enabling efficient and specific delivery of therapeutic miRNAs. Preclinical studies in cardiovascular diseases have shown that exosomes can transport bioactive molecules, reduce apoptosis, modulate inflammatory pathways, and promote tissue repair [102]. These features highlight their potential in oncology, where they could be engineered to carry tumor-suppressive miRNAs or oncomiR inhibitors, thereby improving biodistribution and therapeutic efficacy.

### 4.5. Polymer–Drug Conjugates (PDCs)

In oncology, polymer–drug conjugates (PDCs) represent a specialized class of nanocarriers within polymer-based delivery strategies, offering improvements in solubility, controlled release, and pharmacokinetic profiles. Advances in controlled polymerization and examples such as PEG–doxorubicin have demonstrated enhanced efficacy and reduced toxicity. Moreover, incorporating targeting ligands enables site-specific release, protecting therapeutic agents and improving selectivity, with applications in cancer, metastasis, and other diseases [103]. Although PDCs classically carry small molecules, hybrid constructs (polymer–miRNA or polymer–ligand–miRNA) can couple controlled release with active targeting, and co-deliver oncomiR inhibitors with chemotherapy to overcome resistance [103,104,105].

### 4.6. Other Nanotechnologies (Polymersomes, Nanocapsules, Nanoemulsions)

Other nanotechnology-based systems have also demonstrated the ability to enhance the bioavailability and therapeutic performance of bioactive molecules. Examples include targeted polymersomes, nanocapsules, and protein-based nanoparticles, which have improved central nervous system delivery, as well as surface-modified nanoparticles, alginate nanocomposites, and nanoemulsions, which have shown increased stability and extended therapeutic effects in disease models. Although developed for compounds such as curcumin, these designs provide valuable insights for adapting delivery strategies to miRNA-based cancer therapies [106]. Polymersomes, nanocapsules, and nanoemulsions can be engineered for CNS (central nervous system) delivery by improving penetration of the BBB (blood–brain barrier) and brain tumor interfaces, enabling targeted payload transport and controlled release [107]. In parallel, these platforms support imaging-guided theranostics, combining therapy and diagnostics, using MRI (magnetic resonance imaging) contrast or fluorescent labels to noninvasively track biodistribution and tumor uptake in vivo [108]. Early and contemporary reports show polymersomes can co-load drugs and imaging agents for MR/optical tracking, while lipid nanocapsules and nanoemulsions have been adapted for brain delivery and visualization of accumulation within brain tumors [109].

### 4.7. Safety, Immunogenicity, and Regulatory Considerations

Innate immune sensing remains a key safety determinant for RNA therapeutics: single-stranded RNA can activate TLR7/8 (Toll-like receptor 7/8) unless mitigated by nucleoside chemistry (e.g., 2′-O-methyl, 2′-F, LNA), which dampens interferon responses while preserving activity [110,111,112]. Complement activation, classically described as CARPA (complement activation-related pseudo-allergy), and anti-PEG responses can also drive acute hypersensitivity and altered exposure, especially with PEGylated or lipidic carriers [113]. In parallel, hepatic and splenic uptake by the RES (reticuloendothelial system) concentrates the dose in the liver/spleen, shaping both efficacy and toxicity profiles [114]. Together, these factors argue for careful sequence design plus carrier composition (ionizable lipids, helper lipids, PEG-lipids) and preclinical immunotoxicology screens [115].

From a quality and regulatory perspective, nanocarrier-based miRNA products must control CQAs (critical quality attributes), including particle size/PDI (polydispersity index), zeta potential, encapsulation efficiency, release kinetics, endotoxin, sterility, and stability; route of administration (i.v. = intravenous; intratumoral; inhaled) and repeat dosing materially influence PK/PD and safety readouts [116,117]. FDA guidance for drug products containing nanomaterials provides expectations for characterization and risk assessment across these domains [118]. Clinically, the liposomal miR-34a mimic (MRX34) Phase I was halted early due to immune-mediated toxicities [67], underscoring these risks, whereas the miR-16 mimic (TargomiR/MesomiR-1) showed acceptable tolerability in a Phase I mesothelioma/NSCLC study, differences that likely reflect sequence, carrier, dose/schedule, and patient context [68].

From a development standpoint, a first-in-human miRNA nanocarrier program benefits from a prespecified pathway that links CMC, safety monitoring, and pharmacology. Starting-dose selection is typically anchored to the nonclinical safety package and MRSD principles, with conservative escalation and clear stopping rules when innate immune activation is a plausible risk. In practice, escalation designs often incorporate sentinel dosing and step-up approaches, together with premedication and infusion-rate management when hypersensitivity is anticipated. Immunomonitoring can be streamlined but should be mechanism-aware, commonly including cytokine panels (e.g., IL-6, TNF-α), complement activation readouts (e.g., C3a/C5a or sC5b-9), and anti-PEG/anti-carrier antibodies when applicable, alongside routine hematology, liver enzymes, and coagulation markers. Biomarker plans should prioritize fit-for-purpose evidence of target engagement and exposure–response, such as circulating miRNA pharmacokinetics, changes in validated downstream transcripts/proteins in accessible samples (or tumor biopsies when feasible), and exploratory immune markers aligned to the carrier’s risk profile. Collectively, these elements help justify dose selection and support a realistic translation from preclinical performance to early clinical testing.

In sum, chemistry + carrier + targeting + endosomal escape determine clinical performance of miRNA therapeutics, and combination strategies (mimic or inhibitor plus standard therapy) are poised to address resistance and heterogeneity in solid tumors [90,91,119].

## 5. Applications of miRNAs Beyond Cancer

Although this review focuses on oncology, miRNA dysregulation also contributes to a broad spectrum of non-malignant diseases, supporting the use of miRNA mimics to restore function or inhibitors (antimiRs) to [120,121] suppress pathogenic activity. Beyond cancer, representative examples highlight transferable development lessons rather than disease-by-disease detail: circulating miRNAs linked to platelet activity and inflammatory mediators may inform cardiovascular risk stratification [122,123]; mechanistic axes such as miR-153–KPNA5–NRF2 illustrate how miRNA modulation can impact neurodegenerative or hypoperfusion [124,125]-related cognitive decline; and extracellular-vesicle-associated miRNAs are implicated in fibrotic [126] remodeling pathways, suggesting that delivery concepts developed in oncology may be adapted to [127] chronic disorders where tissue exposure and safety remain key constraints.

## 6. Representative Examples Across Cancer Types

In 2025, it is estimated that approximately 19 million new cancer cases will be diagnosed worldwide, with 10 million deaths. Projections indicate that by 2050, these numbers could exceed 33 million cases and 18 million deaths, driven mainly by global population growth and aging [128]. They have been explored as potential therapeutic targets showing promise in breast, lung, pancreatic, and ovarian cancer [129], among others. Within this landscape, tumor-specific microRNA programs enable biomarker discovery and therapeutic targeting across malignancies.

### 6.1. miRNAs in Breast Cancer

Breast cancer remains the most common malignancy among women and is classified into subtypes according to molecular profiles. Its tumor progression and metastasis have been linked to miRNA dysregulation, characterized by the overexpression of oncomiRs and the downregulation of tumor-suppressor miRNAs, promoting processes such as angiogenesis, epithelial–mesenchymal transition (EMT), and adipose tissue remodeling [130].

miRNAs also function as biomarkers in the pathogenesis of breast cancer. A recent study proposed a theranostic strategy using Ce6-DNAzyme@ZIF-8@PEG nanoparticles, which amplify the miR-21 signal and enhance photodynamic therapy (PDT) by inhibiting reactive oxygen species (ROS) scavenging. In vivo preclinical results demonstrated that this strategy increased PDT efficiency, leading to significant tumor inhibition [131]. A microarray analysis (758 miRNAs) revealed significant changes in a panel of miRNAs related to breast cancer, including miR-10a-5p, miR-322-5p, miR-450a-5p, miR-142-5p, miR-148b-3p, miR-18a-5p, and miR-347 (notably, miR-322-5p is commonly used for the rodent ortholog of hsa-miR-424-5p, and miR-347 is predominantly reported in rodent annotations; therefore, both entries should be interpreted as species-specific and independently validated in human cohorts, using explicit species notation where applicable). These results suggest that the regulation of these miRNAs could influence oncogenesis and tumor suppression in breast cancer. In preclinical models, Seaberry (*Hippophae rhamnoides* L.) fruit peel extracts were shown to affect the expression of these miRNAs, reinforcing their exploratory therapeutic potential in the treatment of breast cancer [132].

In breast cancer, the tumor-suppressive let-7 miRNA is often reduced, largely due to inhibition by the RNA-binding protein Lin28. This dysregulation promotes proliferation, invasion, and metastasis. Restoring let-7 levels by targeting Lin28 offers a promising therapeutic approach [133]. miR-21/let-7/miR-200/miR-155 orchestrate proliferation, EMT, and immunity; preclinical theranostic approaches and biomarker panels show promise pending robust clinical validation.

### 6.2. miRNAs in Papillary Thyroid Cancer

In papillary thyroid cancer (PTC), miR-7-5p is significantly downregulated and directly targets EGFR/IRS2, attenuating MAPK/PI3K signaling; restoring miR-7-5p reduces proliferation and sensitizes to pathway inhibitors. Emerging data also link miR-7-5p to p53 stability via the IPO7 axis, suggesting dual control of growth and stress responses. Reports of pro-oncogenic effects appear model-dependent and should be interpreted within specific experimental contexts [134,135].

### 6.3. miRNAs in Hepatocellular Carcinoma

In HCC cell models, oncogenic miR-222 can promote LINE-1 retrotransposition, potentially fueling genomic heterogeneity; this effect is partly associated with reduced let-7c activity. While mechanistic work is ongoing, these findings reinforce miR-222 as a multifunctional driver in HCC and nominate retrotransposition control as a translational vulnerability [136].

Non-coding RNA cross-talk can be understood as an integrated regulatory network in which miRNAs act as central post-transcriptional repressors, while other RNA species, such as circRNAs and lncRNAs, modulate miRNA availability and activity. In many settings, circRNAs/lncRNAs behave as competing endogenous RNAs by sequestering specific miRNAs (“sponging”), which can relieve repression of shared mRNA targets and shift downstream protein programs. Because these relationships are often context-dependent, strong evidence typically goes beyond expression correlations and includes binding-site validation, perturbation and rescue experiments, and consistent effects on the corresponding mRNA/protein axis. The following examples are presented as representative instances of this general cross-talk architecture.

### 6.4. miRNAs in Gastric Cancer: Regulation via circRNA Interactions

In gastric cancer, multiple circRNA–miRNA–mRNA axes modulate miRNA availability and downstream gene programs involved in proliferation, invasion, metabolism, and therapy resistance, thereby reinforcing the central role of miRNA control in disease progression and treatment [137] response. These networks are increasingly viewed as biomarker and intervention opportunities [138,139,140] when supported by mechanistic validation. For example, circNRIP1 can sponge miR-149-5p and thereby influence AKT1/mTOR signaling, illustrating how circRNA hubs may reshape miRNA-mediated regulation of oncogenic pathways and chemoresistance.

### 6.5. miRNAs and Cervical Cancer: The miR-16-5p–FASN Axis

In cervical cancer, Linc00662 sponges miR-16-5p to de-repress FASN, promoting proliferation, migration, and invasion; miR-16-5p restoration or Linc00662 inhibition reverses these phenotypes, supporting a lipid-metabolism vulnerability [141]. This lncRNA–miRNA–mRNA axis exemplifies ceRNA-like cross-talk in which miRNA sequestration rewires metabolic outputs in a tumor-context-dependent manner.

### 6.6. Areca Nut-Associated miRNA Dysregulation in Head and Neck Cancer

In HNC, areca nut consumption (Group 1 carcinogen) is associated with miRNA alterations linked to migration, invasion, ROS production, and stem-like traits. Profiling has identified signatures that include miR-499, connected to oncogenic programs under exposure. However, functional data indicate miR-499a-5p can act as a tumor-suppressive brake on areca-induced migration, invasion, and chemoresistance, highlighting context-dependent roles and motivating joint miRNA–mRNA analyses to uncover actionable targets [142,143].

### 6.7. HPV-Related Cancers

miRNAs are promising biomarkers in HPV-related cancers (see Table 5). Recurrent alterations include upregulation of miR-16/21/25/92a/378 and downregulation of miR-22/27a/29a/100 across cohorts. Multi-miRNA panels, e.g., miR-20a, miR-92a, miR-141, miR-183-5p, miR-210, miR-944, enhance diagnostic performance. In p16-positive OPSCC, overexpression of miR-182-5p, miR-205-5p, miR-133a-3p, miR-155 stratifies prognosis, supporting panel-based screening and risk assessment [144,145,146,147].

### 6.8. Concluding Remarks

Tumor-specific microRNA programs converge on core oncogenic processes, proliferation, EMT, immunity, stress responses, and metabolism, while emerging delivery/theranostic strategies and multi-miRNA panels move toward clinical translation. Integrating model-dependent signals (e.g., miR-7-5p in PTC), retrotransposition control (miR-222/LINE-1 in HCC), and circRNA hubs (e.g., circNRIP1 in GC) outlines actionable paths for biomarker development and targeted interventions. Immediate priorities include multicenter validation of panels, standardized nomenclature (locus/arm, 5p/3p), and preclinical trials that link mechanisms to clinical endpoints.

## 7. Challenges and Limitations

Challenges remain, including the development of efficient delivery systems, stabilization of nucleic acids, and reduction in potential adverse effects, which still limit clinical application [148]. Additional hurdles include biodistribution and tumor penetration, endosomal escape, off-target repression and seed-mediated interactions, immunostimulation (TLR activation), manufacturing scale-up and reproducibility, and the lack of harmonized potency/quality assays for regulatory approval. Addressing these issues will require standardized analytics, robust in vivo models, and head-to-head comparisons of delivery platforms. Action items to accelerate clinical translation. To address the main barriers identified above, we propose a focused set of practical priorities for the field. Standardize analytical workflows that capture isomiRs and related sequence variants and quantify target engagement with reproducible reporting. Harmonize potency and comparability assays across platforms, using fit-for-purpose readouts that link miRNA modulation to downstream mRNA/protein effects. Establish clinically validated extracellular vesicle workflows (isolation, normalization, cargo quantification, and reporting) to improve reproducibility and interpretation. Manage immunogenicity risk proactively, including formulation-dependent innate immune activation testing and clinically relevant immunomonitoring during dose escalation. Finally, design rational combination strategies (including standard-of-care agents) using biomarker-guided dosing, appropriate controls, and delivery systems matched to the intended tissue exposure and safety constraints.

## 8. Future Perspectives

Nucleic acid-based therapies have become a major advance in biomedicine and [149] already support clinical strategies in genetic disorders and cancer, contributing to more personalized approaches aimed at improving efficacy while limiting adverse effects. In the near term, further progress will likely depend on integrating well-characterized delivery platforms, including nanotechnologies, with a clearer understanding of cellular mechanisms [150] that shape biodistribution, target engagement, and safety. Alongside these evidence-supported directions, emerging findings from circRNA research suggest that circRNAs can influence miRNA activity and oxidative-stress responses, raising the possibility that redox modulation could reshape non-coding RNA networks. However, the extent to which natural antioxidants (e.g., quercetin, curcumin, resveratrol, astaxanthin) can reproducibly and clinically modulate these pathways remains to be established. Conceptually, combining miRNA therapeutics with redox modulators and co-delivery strategies may improve efficacy and tolerability, but this remains hypothesis-generating and requires rigorous in vivo validation. Priorities therefore include robust preclinical testing, redox–miRNA profiling to inform patient selection, and addressing bioavailability and pharmacokinetic constraints [151].

## 9. Conclusions

MicroRNA-centered strategies are converging with advances in nanodelivery, chemistry, and systems profiling to translate mechanistic insights into clinical value. Across tumors, miRNAs integrate proliferation, EMT, immunity, stress responses, and metabolism; panel-based diagnostics and theranostic approaches are progressing, yet delivery, specificity, and safety remain the main bottlenecks. Short-term priorities include multicenter validation of multi-miRNA signatures, standardized nomenclature (locus/arm; 5p/3p), and preclinical studies that link endosomal escape and biodistribution to pharmacodynamic readouts. In the medium term, co-delivery designs (miRNA agents plus redox modulators) and circRNA-informed targeting may improve efficacy–toxicity balance. Overall, aligning analytical rigor with rational combination therapy will be key to moving miRNA therapeutics from promise to practice. A practical way forward is to prioritize standardized isomiR-aware analytics, harmonized potency/comparability assays, clinically validated EV workflows, proactive immunogenicity monitoring, and biomarker-guided rational combination studies.

## Figures and Tables

**Table 1:** Quick reference for specialized terms used in miRNA biogenesis and turnover.

Term Used in the Text	What It Is (Simple Definition)	Why It Matters (Therapeutic/Oncology Relevance)	Manuscript Location	References
Microprocessor (DROSHA–DGCR8)	Nuclear complex that cleaves pri-miRNAs into pre-miRNAs	Determines mature miRNA output; altered processing can reshape oncogenic/tumor-suppressive programs	Section 2, opening biogenesis paragraph	[19,20,21,22,23]
Basal UG/downstream CNNC/lower-stem mGHG motifs	Short pri-miRNA sequence/structure cues that can improve Drosha cleavage precision and efficiency	Helps interpret processing variability, microheterogeneity, and functional shifts	Section 2, opening biogenesis paragraph	[20,21,22,23,24]
Lower-stem loops/bulges (“lower-stem defects”)	Local structural features that can reduce productive cropping or increase alternative cuts	Can shift mature miRNA yield and isoform distribution; relevant to context-dependent outputs	Section 2, opening biogenesis paragraph	[24,25]
RNA-binding proteins (e.g., SRSF7/SRSF3)	Proteins that modulate pri-miRNA processing by influencing Microprocessor recruitment/efficiency	Adds cell-type specificity; contributes to context dependence in cancer	Section 2, opening biogenesis paragraph	[26]
DGCR8-responsive element (DRE)	RNA element that increases DGCR8 sensitivity for specific pri-miRNAs	Highlights substrate selectivity; not all pri-miRNAs are processed equally	Section 2, DGCR8 paragraph	[27]
IsomiRs	miRNA variants generated by alternative cleavage and/or 3′ modifications	Can alter targeting and stability; important for sequencing interpretation and therapeutic design	Section 2, end of DGCR8 paragraph	[28,29]
A-to-I editing (ADAR1/ADAR2)	Adenosine-to-inosine RNA editing that can change processing and/or retarget a miRNA	May rewire targets and contribute to cancer-associated regulation	Section 2, end of DGCR8 paragraph	[11,30]
miR-451 (Dicer-independent maturation)	miRNA processed by AGO2 slicing and PARN trimming (with CSDE1 support), bypassing Dicer	Illustrates non-canonical maturation; relevant when discussing pathway constraints	Section 2.1 Non-Canonical Pathways	[31]
Mirtrons	Splicing-derived pre-miRNA-like hairpins that bypass Drosha cropping	Expands biogenesis routes; may be regulated differently across tissues and disease	Section 2.1 Non-Canonical Pathways	[32,33]
rG4 checkpoint in mirtrons	RNA G-quadruplex structure that can modulate mirtron splicing/maturation	Adds a structural “gate” controlling small-RNA output	Section 2.1 Non-Canonical Pathways	[33]
sdRNA/tsRNA (small RNA fragments)	Small RNAs derived from other RNA classes (e.g., snoRNAs/tRNAs) that may show miRNA-like behavior in some contexts	Prevents confusion with miRNAs and clarifies scope when discussing broader small-RNA layers	Section 2 (small-RNA discussion)	[34]

A-to-I—adenosine-to-inosine; ADAR—adenosine deaminase acting on RNA; AGO2—Argonaute 2; CSDE1—cold shock domain containing E1; DGCR8—DiGeorge syndrome critical region 8; DROSHA—ribonuclease III Drosha; miRNA—microRNA; PARN—poly(A)-specific ribonuclease; rG4—RNA G-quadruplex; sdRNA—snoRNA-derived RNA; snoRNA—small nucleolar RNA; tsRNA—tRNA-derived small RNA; tRNA—transfer RNA.

**Table 2:** Representative oncogenic and tumor-suppressive microRNAs in cancer.

Functional Role	Representative miRNA(s)	Validated Targets/Pathways (Examples)	Main Tumorigenic Processes	Cancer Examples	Reference
Oncogenic (oncomiR)	miR-21	PTEN, PDCD4; PI3K/AKT	Proliferation, invasion, survival	Breast, lung, colorectal, GBM	[53]
Oncogenic (oncomiR)	miR-155	SOCS1, SHIP1, TP53INP1; immune/TME	Proliferation, immune evasion, angiogenesis	Lymphoma, breast, cervical	[54]
Oncogenic (metastasis)	miR-10b	HOXD10 → RhoC	EMT, invasion, metastasis	Breast, colorectal, ovarian	[55]
Oncogenic (oncomiR)	miR-221/222	p27Kip1 (CDKN1B), PTEN, TIMP3	Proliferation, invasion	HCC, thyroid, breast	[56]
Tumor suppressor	miR-34a (miR-34a-5p)	p53 network; MET, CDK6, BCL2	Cell-cycle arrest, apoptosis, anti-metastasis	Lung, HCC, breast, colorectal	[57]
Tumor suppressor	let-7 family	RAS, HMGA2 (LIN28/let-7 axis)	Anti-proliferation, anti-EMT	Lung, brain, multiple	[58]
Tumor suppressor	miR-15a/16-1	BCL2 (apoptosis)	Apoptosis, anti-survival	CLL, prostate	[59]
Tumor suppressor	miR-143/145	KRAS/MAPK; cytoskeleton/actin programs	Anti-invasion, stromal restraint	Colorectal, GI cancers	[60]
Tumor suppressor (anti-EMT)	miR-200 family	ZEB1/ZEB2; EMT core loop	EMT suppression, anti-metastasis	Breast, lung, brain mets	[61]

EMT—epithelial–mesenchymal transition; TME—tumor microenvironment; GBM—glioblastoma; HCC—hepatocellular carcinoma; CLL—chronic lymphocytic leukemia; GI—gastrointestinal. Nomenclature note: “p27Kip1” refers to the protein encoded by CDKN1B. In this manuscript, “miR-34a” denotes the 5p strand.

**Table 3:** MicroRNAs shaping the tumor microenvironment (TME).

miRNA	Source/Cell Type	Predominant Effect in TME	Axis/Pathway (as Supported in Text/Refs)	Cancer Context (Examples)	Reference
miR-155	Tumor-associated macrophage (TAM)	Modulates macrophage polarization (M1/M2); shifts anti/pro-tumor programs	Macrophage polarization (M1/M2)	Various cancers (review)	[62]
miR-21	Tumor cell/immune (regulatory role)	Immunoregulatory; contributes to macrophage polarization (M1/M2)	Macrophage polarization (M1/M2)	Various cancers (review)	[62]
miR-146a	Myeloid/immune cells	Immunoregulatory; contributes to macrophage polarization (M1/M2)	Macrophage polarization (M1/M2)	Various cancers (review)	[62]
miR-1246 (exosomal)	Tumor cell → immune cells	PD-L1 stabilization; CD8^+^ T-cell suppression; immune evasion	PD-L1 (stability/immune checkpoint); links to angiogenesis/EMT described in review	Various cancers (review)	[63]
miR-21-5p (exosomal)	M2 macrophages → tumor cells	Increased invasion; therapy resistance	PTEN → AKT signaling	Renal cell carcinoma (study)	[64]

TME—tumor microenvironment; TAM—tumor-associated macrophage; miRNA—microRNA; EMT—epithelial–mesenchymal transition; PD-L1—programmed death-ligand 1; CD8^+^—cluster of differentiation 8-positive T cells; PTEN—phosphatase and tensin homolog; AKT—protein kinase B; M1/M2—classically activated (pro-inflammatory)/alternatively activated (immunoregulatory) macrophage states; exosomal—extracellular-vesicle derived. Symbols: “→” indicates directionality/causal flow in signaling (e.g., upstream factor leading to downstream activation/effect).

**Table 4:** Clinical-stage microRNA therapeutics discussed in this review: trial phase, indication, delivery platform/route, key chemical modifications, and reported outcomes/limitations.

Program	Therapeutic Modality	Delivery Platform/Route	Key Chemical Modifications	Indication (Example)	Phase/Design (High Level)	Primary Endpoint(s) (High Level)	Clinical Outcomes & Key Limitations	Reference
MRX34	miR-34a mimic (liposomal formulation)	Liposomal NP; IV infusion	miR-34a mimic; chemical modifications not publicly disclosed	Advanced solid tumors (incl. HCC)	Phase I, dose escalation	Safety/tolerability (DLT/MTD/RP2D); PK/PD; preliminary activity	Trial stopped early due to immune-mediated SAEs (incl. deaths); limited tolerability	[67]
TargomiR/MesomiR-1	miR-16 family mimic loaded in EGFR-targeted minicells	EGFR-targeted minicells (EDVs); IV	miR-16 family mimic; chemical modifications not publicly disclosed	Malignant pleural mesothelioma	Phase I, open-label, 3 + 3 dose escalation	Safety (DLT/MTD), optimal dosing frequency; preliminary activity	MTD established; infusion-related inflammatory toxicities; PR/SD signals reported	[68]
Cobomarsen (MRG-106)	anti-miR-155 inhibitor (LNA-based oligonucleotide)	Systemic or lesion-directed (per protocol)	LNA-modified anti-miR-155	CTCL/mycosis fungoides	Phase II, randomized, open-label, active comparator	Efficacy and safety (e.g., ORR + safety)	Program terminated (business/prioritization); activity/safety reported in CTCL	[69]
Miravirsen (SPC3649)	anti-miR-122 (LNA antimiR)	Subcutaneous injection	LNA phosphorothioate anti-miR-122	Chronic HCV infection	Phase IIa, randomized, placebo-controlled, dose-escalation	Antiviral activity (HCV RNA decline) + safety	Dose-dependent reduction in HCV RNA; informs antimiR chemistry/safety	[70]
Lademirsen (RG-012)	anti-miR-21 oligonucleotide	Subcutaneous injection	anti-miR-21; chemical modifications not publicly disclosed	Alport syndrome	Phase II, randomized, double-blind, placebo-controlled (then open-label)	Kidney function decline (e.g., eGFR slope) + safety	Generally well-tolerated; no meaningful improvement vs. placebo; terminated for futility	[71]
AMT-130	AAV5 gene therapy expressing engineered miRNA targeting HTT (miHTT)	AAV5; intrastriatal neurosurgical delivery	vectorized artificial miRNA (miHTT)	Huntington’s disease	Phase I/II, first-in-human (sham-controlled cohorts + open-label)	Safety/PoC; disease progression measures	Ongoing program; topline results reported by the sponsor (not yet peer-reviewed).	[72]

AAV—adeno-associated virus; SAE—serious adverse event; CTCL—cutaneous T-cell lymphoma; DLT—dose-limiting toxicity; eGFR—estimated glomerular filtration rate; EGFR—epidermal growth factor receptor; HCC—hepatocellular carcinoma; HCV—hepatitis C virus; LNA—locked nucleic acid; MTD—maximum tolerated dose; ORR—objective response rate; PD—pharmacodynamics; PK—pharmacokinetics; PoC—proof-of-concept; PR—partial response; RP2D—recommended phase 2 dose; SD—stable disease.

**Table 5:** Summary of tumor-specific microRNA examples across cancer types, highlighting key targets, biological processes, evidence tier, and translational notes.

Cancer Type	Key miRNAs (Examples)	Main Targets/Axis	Process	Evidence	Translational Note	References
Breast	miR-21, let-7, miR-200, miR-155; panel (miR-10a-5p, miR-18a-5p, miR-142-5p, miR-148b-3p, miR-322-5p/424-5p ^a^, miR-347 ^a^, miR-450a-5p ^a^)	PTEN/PDCD4; LIN28–let-7; EMT network	Proliferation, EMT, immunity	Preclinical + cohorts	Theranostics (PDT); circulating/panel biomarkers pending validation	[130,131,132,133]
Papillary thyroid (PTC)	miR-7-5p (downregulated)	EGFR/IRS2; IPO7–p53	MAPK/PI3K tuning; p53 stability	Cells + datasets	Model-dependent effects; sensitizes to pathway inhibitors	[134,135]
Hepatocellular (HCC)	miR-222 (upregulated)	LINE-1 retrotransposition; let-7c (reduced)	Genomic heterogeneity	Cell models	Retrotransposition control as vulnerability	[136]
Gastric (GC)	circNRIP1 → miR-149-5p → AKT1/mTOR (example axis)	AKT1/mTOR	Chemoresistance, EMT	Preclinical/cohorts	circRNAs as biomarkers and targets	[137,138,139,140]
Cervical	Linc00662–miR-16-5p–FASN	FASN	Lipid metabolism, invasion	Cells/tissues	Restore miR-16-5p or inhibit Linc00662	[141]
Head & Neck (areca)	miR-499/miR-499a-5p ^a^	—	Migration, invasion, ROS, stem-like traits	Profiling + functional	Context-dependent roles; joint miRNA–mRNA analyses	[142,143]
HPV-related	miR-16/21/25/92a/378 (up); miR-22/27a/29a/100 (down); panels (miR-20a/92a/141/183-5p/210/944)	—	Screening, prognosis	Cohorts	Add AUROC/sensitivity/specificity when available	[144,145,146,147]

AKT1—RAC-alpha serine/threonine-protein kinase; AUROC—area under the receiver-operating characteristic curve; circRNA—circular RNA; EGFR—epidermal growth factor receptor; EMT—epithelial–mesenchymal transition; FASN—fatty acid synthase; GC—gastric cancer; HCC—hepatocellular carcinoma; IPO7—importin-7; IRS2—insulin receptor substrate 2; LINE-1—long interspersed nuclear element-1; MAPK—mitogen-activated protein kinase; miRNA—microRNA; mRNA—messenger RNA; mTOR—mechanistic target of rapamycin; PDT—photodynamic therapy; PI3K—phosphoinositide 3-kinase; PTC—papillary thyroid cancer; ROS—reactive oxygen species. Symbols: → denotes direction/activation in regulatory axes; /indicates paired targets/pathways. ^a^ Nomenclature note: “miR-499” in profiling commonly labels the family; functional assays referenced here used miR-499a-5p; miR-322-5p and miR-450a-5p are commonly used for the rodent ortholog of hsa-miR-424-5p; miR-347 is predominantly annotated in rodents. When discussed in a human context, entries should be reported with appropriate species notation and validated in human datasets.

## Data Availability

No new data were created or analyzed in this study. Data sharing is not applicable to this article.

## Abbreviations

The following abbreviations are used in this manuscript:

AGO	Argonaute
ADAR	Adenosine deaminase acting on RNA
AKT	Protein kinase B
AKT1	RAC-alpha serine/threonine-protein kinase
AUROC	Area under the receiver-operating characteristic curve
BBB	Blood–brain barrier
BCL2	B-cell lymphoma 2
CD8^+^	CD8-positive T cells
CDK6	Cyclin-dependent kinase 6
CDKN1B	p27Kip1 (Cyclin-dependent kinase inhibitor 1B)
CDS	Coding sequence
circRNA	Circular RNA
CLIP	Crosslinking immunoprecipitation
CLL	Chronic lymphocytic leukemia
CNS	Central nervous system
DCP1	Decapping enzyme 1
DCP2	Decapping enzyme 2
DGCR8	DiGeorge critical region 8
Dicer	RNase III endonuclease Dicer
DROSHA	RNase III endonuclease DROSHA
DNAzyme	Deoxyribozyme
EGFR	Epidermal growth factor receptor
EMT	Epithelial–mesenchymal transition
EVs	Extracellular vesicles
FASN	Fatty acid synthase
GalNAc	N-acetylgalactosamine
GBM	Glioblastoma
GI	Gastrointestinal
GW182	GW182 protein family
HCC	Hepatocellular carcinoma
HMGA2	High mobility group AT-hook 2
HNC	Head and neck cancer
HOXD10	Homeobox D10
Hsc70	Heat shock cognate 70
Hsp90	Heat shock protein 90
IPO7	Importin-7
IRS2	Insulin receptor substrate 2
i.v.	Intravenous
LINE-1	Long interspersed nuclear element-1
LNA	Locked nucleic acid
LNPs	Lipid nanoparticles
lncRNA	Long non-coding RNA
MAPK	Mitogen-activated protein kinase
MET	MET proto-oncogene (HGFR)
miRNA	MicroRNA
miRISC/RISC	miRNA-induced silencing complex/RNA-induced silencing complex
MRX34	miR-34a mimic (clinical program)
mRNA	Messenger RNA
mTOR	Mechanistic target of rapamycin
NLCs	Nanostructured lipid carriers
OPSCC	Oropharyngeal squamous cell carcinoma
PACT	Protein activator of PKR (PACT)
PAN2–PAN3	PAN2–PAN3 deadenylase complex
PARN	Poly(A)-specific ribonuclease
PDCs	Polymer–drug conjugates
PD	Pharmacodynamics
PD-1	Programmed cell death protein 1
PD-L1	Programmed death-ligand 1
PDCD4	Programmed cell death 4
PEG	Polyethylene glycol
PEGylated	PEG-modified
PI3K	Phosphoinositide 3-kinase
PK	Pharmacokinetics
PLGA	Poly(lactic-co-glycolic acid)
pre-miRNA	Precursor microRNA
pri-miRNA	Primary microRNA
PTC	Papillary thyroid cancer
PTEN	Phosphatase and tensin homolog
RAS	Rat sarcoma (small GTPase)
RES	Reticuloendothelial system
rG4	RNA G-quadruplex
RhoC	Ras homolog family member C
RTP	Retrotransposition
sdRNA	snoRNA-derived RNA
SHIP1	SH2-containing inositol phosphatase 1
SLNs	Solid lipid nanoparticles
SOCS1	Suppressor of cytokine signaling 1
TDMD	Target-directed miRNA degradation
TIMP3	Tissue inhibitor of metalloproteinases 3
TLA	Three-letter acronym (placeholder; remove if not used)
TME	Tumor microenvironment
TNRC6	TNRC6 (GW182) protein family
TRBP	TAR RNA-binding protein
tsRNA	tRNA-derived small RNA
tRF	tRNA-derived fragment
UTR	Untranslated region
VEGF	Vascular endothelial growth factor
ZEB1	Zinc-finger E-box-binding homeobox 1
ZEB2	Zinc-finger E-box-binding homeobox 2
ZIF-8	Zeolitic imidazolate framework-8
